# Gravity-driven flow for intracranial saccular aneurysm filling

**DOI:** 10.1098/rsif.2025.0968

**Published:** 2026-10-28

**Authors:** Tirosh Mekler, Maria Khoury, Yevgeniy Kreinin, Rami Fishler, Nir Natan, Ron Bessler, Alexander Leshansky, Josué Sznitman, Netanel Korin

**Affiliations:** ^1^ Department of Biomedical Engineering, Technion—Israel Institute of Technology, Haifa, Israel; ^2^ Department of Chemical Engineering, Technion—Israel Institute of Technology, Haifa, Israel; ^3^ Fischell Department of Bioengineering, University of Maryland, College Park, MD, USA

**Keywords:** intracranial aneurysm, gravity-driven flow, computational fluid dynamics, injectable biomaterials, aneurysm embolization

## Abstract

Intracranial aneurysms (IAs) are life-threatening cerebrovascular malformations in which complete and durable sac closure is the primary goal of endovascular treatments, including coiling, stent-assisted coiling and flow-diverter implantation. However, standard catheter-based approaches may fail to fully occlude the sac and are limited by the need for direct access. Liquid embolic agents offer better geometric conformity, but their deployment remains constrained by similar access issues. In this study, we present a gravity-driven approach for distal filling of IAs. Through a combination of analytical modelling, *in vitro* experiments with idealized aneurysm models, and patient-specific computational fluid dynamics (CFD) simulations, we demonstrate that efficient filling of aneurysm cavities from relatively remote locations may be achieved using a heavier injectable fluid, while properly adjusting tilt angle and injection flow rate. A phase diagram of flow regimes highlights the interplay between gravitational, inertial and viscous forces critical to successful filling. Our findings show that slow, gravity-assisted injections minimize bypass and spillover, enabling targeted aneurysm filling even in complex geometries. CFD simulations highlight the need for patient-specific planning of such a procedure accounting for personal vascular anatomy. This strategy may provide a safer and less invasive alternative to current endovascular techniques, especially for anatomically inaccessible aneurysms.

## 1. Introduction

Intracranial aneurysms (IAs) result from weakened cerebral arterial walls, forming bulges prone to rupture and bleeding. IAs are typically located near bifurcations [1,2]. Rupture may occur when the structural integrity of the aneurysm no longer withstands the physiological forces exerted upon it. The rupture of cerebral aneurysms accounts for 70%–85% of non-traumatic subarachnoid haemorrhages (SAH) [3]. To identify aneurysms prone to rupture and requiring clinical attention, angiography is performed using contrast agents [4]. Subsequently, aneurysm size and location become defining factors, with key morphological properties such as neck diameter, parent artery (PA) diameter, aneurysm depth and maximum length considered [5]. Contemporary aneurysm treatments primarily revolve around embolization, involving the use of metallic or polymer embolic agents to occlude the aneurysm volume [6,7]. The most common method is coil embolization [8]. A notable drawback of coiling is its relatively low volumetric occlusion rate (approx. 35%) [9], which may necessitate retreatment owing to compaction and recurrence [3]. As coiling procedures continue to evolve, the field of liquid and gel embolic agents has emerged in response to the challenges faced by coiling in treating wide-neck aneurysms for example. A significant advantage of liquid and gel embolic agents lies in their capability to achieve a comprehensive and uniform fill within the aneurysm void [6]. Moreover, biocompatible embolic agents can promote complete healing of the blood vessel, restoring it to its native form with no residual foreign material. Thus, there is emerging interest in developing improved injectable biomaterials for aneurysm repair.

Additionally, on one hand, current endovascular treatment is based on placing solid implants directly at the aneurysm sites, a procedure that entails further risk and limits the treatment to aneurysms that can be accessed all the way to the aneurysm cavity. Thus, complex aneurysms, often found in deep or critical areas of the cerebral vasculature, are known to be associated with higher risks during endovascular interventions [10]. On the other hand, injectable biomaterials and particulate carriers can offer opportunities for treatment that can be initiated distally from the aneurysm site, where the injectable material can navigate to act at the aneurysm cavity. For instance, in the context of particles, magnetic-based navigation and targeting have been studied widely, as early as in the 1980s [11]. Nevertheless, so far this approach has not translated towards treatment. Furthermore, for injectable biomaterials, fluid-based navigation approaches can potentially navigate the injectable agent further downstream of the catheter tip towards the distal aneurysm cavity site. However, such approaches have not been explored.

Previous research has demonstrated the therapeutic potential of gravity-driven interventions in various medical applications, such as artificial gravity for cardiovascular rehabilitation, lymphatic drainage and vascular pathologies [12]. Additionally, studies have demonstrated that gravity can drive displacement of fluids in cerebral aneurysms, such as in the settling of contrast agents during angiography, attributed to their high density [13]. During intravascular procedures when blood flow is stopped via an upstream balloon, inertial forces are minimal, and such settling effects can become even more pronounced. However, to properly fill the aneurysm, it needs to be oriented in a beneficial direction so that gravity will guide the injected heavier fluid into the cavity (figure 1*a*). Moreover, injection conditions need to be defined as per the properties of the injected fluid, injection site, perfusion rate and parameters of the aneurysm geometry. Building on these observations, this study investigates gravity-driven flow as a new approach for distant filling of intracranial aneurysms with injectable biomaterials, as schematically illustrated in figure 1. Gravity, acting as a passive external force, introduces a controlled and gradual fluid displacement mechanism that can be potentially leveraged to fill aneurysm cavities. This study aimed to identify the optimal conditions for gravity-based filling and its limitations through a combined analytical, experimental and computational framework of the governing flow kinetics and computational fluid dynamics (CFD) simulations of patient-specific models. Altogether, this approach may provide new insights and perspectives on using gravity as a guiding force for the navigation of fluid-based therapeutics to the aneurysm site, potentially reducing procedural risks.

**Figure 1.**
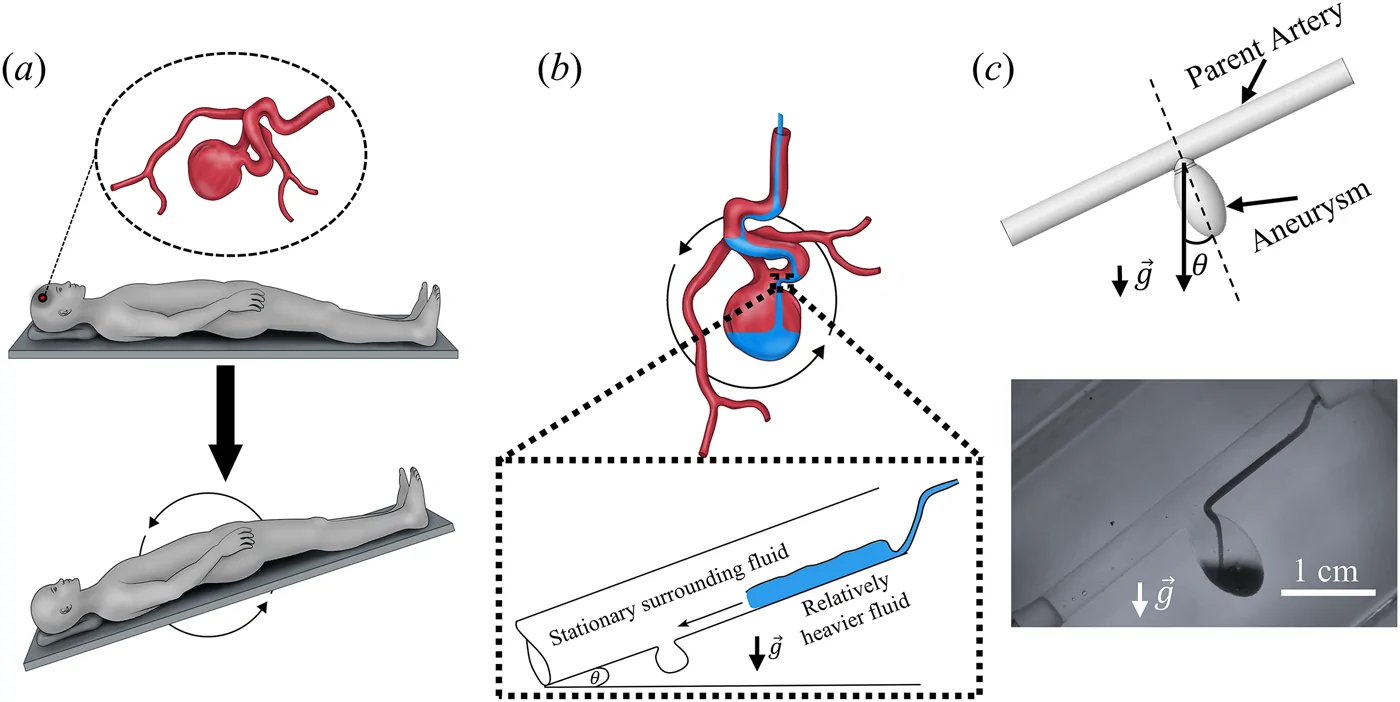
Schematic gravity-driven flow for intracranial saccular aneurysm filling. (*a*) A schematic representation of a patient on a surgery table shows the aneurysm orientation before (upper figure) and after tilting the patient to a gravity-favoured position (lower figure). (*b*) Patient-specific aneurysm after tilting to a gravity-favoured position with a schematic close-up illustrating the fluid flowing downwards by the gravity pull towards the aneurysm. (*c*) Ideal saccular aneurysm model attached to a straight parent artery (PA), 3D-printed for the study (top). Photo from an *in vitro* experiment showing the tilted aneurysm model during the filling procedure (bottom).

## 2. Methods

### 2.1. Gravity-based aneurysm filling—physical mechanism and phase diagram

A simplified analytical model was used to identify the governing dimensionless parameters of gravity-based aneurysm filling and to define the conditions associated with successful and failed filling. The model was intentionally reduced in dimensionality and geometric complexity and was not intended to represent the full three-dimensional aneurysm geometry. Instead, it served as a first-order framework for identifying the dominant physical parameters and constructing a phase diagram for filling behaviour.

Successful filling was defined as entry of the denser injected fluid into the aneurysm cavity without bypassing the opening, whereas failed filling was defined as partial or complete bypass of the aneurysm opening by the injected fluid. Failed filling could also lead to overdose, defined here as continued filling of the parent artery after the aneurysm had reached its maximum filling capacity. The parameters considered in the simplified analysis were the fluid densities (*ρ*) and viscosities (µ), injection flow rate, aneurysm neck diameter (*L_n_*) and tilt angle (*θ*) (figure 2*a*).

**Figure 2.**
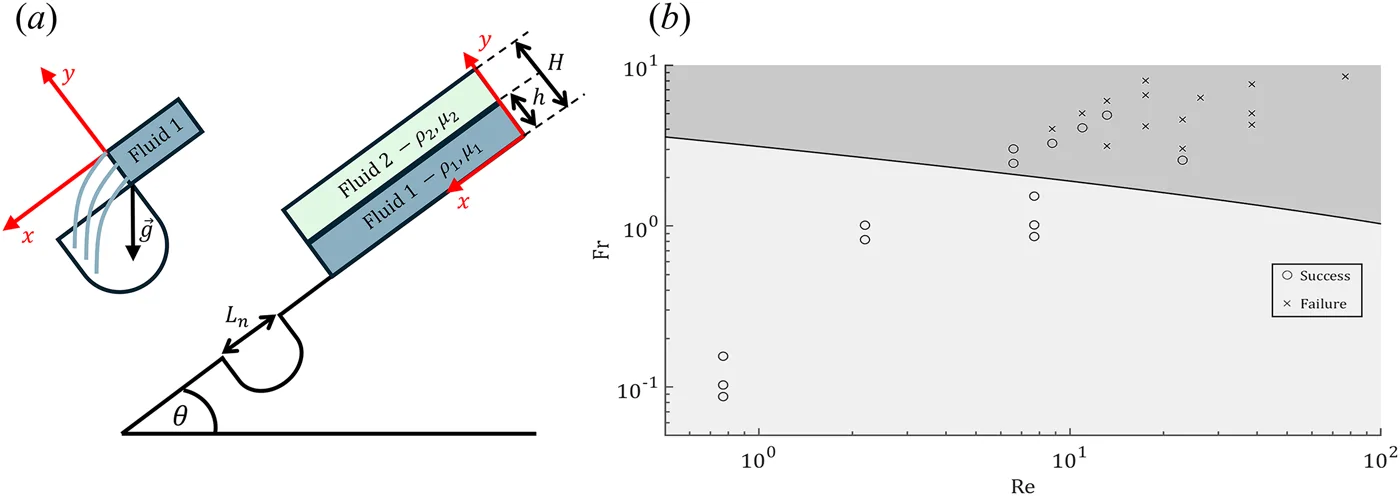
Analytical model schematic and obtained phase diagram. (*a*) Schematic representation of the problem noting the densities (*ρ*_1_ and *ρ*_2_) and the viscosities (µ_1_ and µ_2_) of the injected and the surrounding fluids, respectively. Also, the height of the channel (*H*), the film height (*h*), the length of the aneurysm neck (*L_n_*) and the tilt angle (*θ*). Two additional coordinate systems are shown: one along the slope, used for calculating the flow down the slope, and the other at the tip of the film height of fluid 1 as it reaches the aneurysm cavity, used for calculating the fluid stream trajectory. (*b*) Selected tested cases are shown within Re ranging between 0.1 and 100 and Fr ranging between 0.01 and 10. The experimental cases are symbolized with ‘O’ and ‘X’ and stand for success and failure filling, respectively. The phase diagram was calculated analytically, where the light and dark grey regions stand for successful and failure regions, respectively, for the analytical case of a tilted model at 36° where the solid line divides the two regions.

Based on this framework, the governing dimensionless numbers considered in the analysis were Reynolds (Re) and Froude (Fr) numbers, which characterize the relative importance of inertial forces relative to viscous and gravitational effects, respectively. These parameters were then used to organize the analytical predictions and experimental observations in the phase diagram (figure 2*b*). The Re number was defined as

(2.1) $$Re=\frac{uL}{\nu },$$

where *u* is the average velocity magnitude at a given cross-section, *L* is a characteristic length, taken as the catheter inner diameter (0.58 mm) and ν is the kinematic viscosity, 4.7  ×  10^−7^ m^2^ s^−1,^ corresponding to a 50/50 (v/v) phosphate-buffered saline (PBS)/glycerol mixture. For the theoretical analysis, *u* was calculated from the volumetric flow rate and the analytically determined film height, defined as the thickness of the thin liquid layer flowing downstream towards the aneurysm. For the experimental case, *u* was calculated from the division of the volumetric flow rate by the cross-sectional area of the catheter. A modified Fr number was introduced, which accounts for the difference between the densities of the injected and surrounding fluid, as previously described by Wang *et al.* [13] for evaluating contrast agent settling in cerebral aneurysms, as well as the contribution of the tilt angle,

(2.2) $$Fr=\frac{u}{\sqrt{L_{n}\left(\frac{\rho_{i}-\rho_{c}}{\rho_{c}}\right)g\cos\theta }},$$

where *g* is the gravitational acceleration, *ρ*_*i*_ and *ρ*_*c*_ are the densities of the injected and the surrounding fluid, respectively. In the simplified aneurysm model, *L_n_* was 3.5 mm.

The analytical solution was formulated under the assumptions of a steady-state condition, incompressible flow and two immiscible fluids. The viscous stress of the surrounding (lighter) fluid was neglected. The injected denser fluid was approximated as a gravity-driven laminar film of thickness *h* flowing along an inclined surface with tilt angle *θ* (see supplementary material for the detailed derivation). Under these assumptions, the flow was decomposed into two regimes: (i) gravity-driven film flow along an inclined plane, and (ii) a jet-like flow entering the aneurysm cavity (figure 2*a*). For a region far from the injection site, the flow of the injected fluid was described as a gravity-driven flow of two fluids along an inclined plane. The denser injected fluid occupied the lower region of the channel, adjacent to the lower wall, while the lighter fluid occupied the upper region. Away from the injection site, the flow was assumed to reach steady state with a constant thickness of the denser fluid layer. Under this assumption, mass conservation implied zero volumetric flux of the lighter fluid, corresponding to upstream flow arrest during the clinical procedure done by an upstream-inflated balloon [14]. From these assumptions, the average velocities of the fluids were calculated,

(2.3) $${\bar{V}}_{1}=\frac{gh^{2}\Delta \rho \sin\theta }{3\mu_{i}}\frac{\left(\beta -1\right)\left(\beta +\kappa -1\right)}{\left({\left(\beta -1\right)}^{2}+\kappa \left(4\beta -1\right)\right)},{\bar{V}}_{2}=0,$$

where ${\bar{V}}_{1}$ and ${\bar{V}}_{2}$ are the average velocities at a given cross-section of the injected and surrounding fluids, respectively, *β*  = *H*/*h* > 1 is the ratio of the channel height to the film height and *κ*  = µ_1_/µ_2_ denotes the viscosity ratio between the injected and surrounding fluids. This formulation was developed for a two-dimensional flow, disregarding the side walls and refers to a shallow and wide film (*h* ≪ *H*). The free jet trajectory of the denser fluid into the aneurysm cavity was estimated from the film thickness and velocity obtained from the inclined plane solution, assuming ballistic motion under gravity. Based on the predicted trajectories, cases were classified as successful when the injected fluid entered the aneurysm without bypassing the opening, and as failed when partial or complete bypass occurred. The analytical solution defined regions of successful and failed filling in the phase diagram at a tilt angle of 36°. Experimental results are also shown in figure 2*b*, where successful and failed cases are denoted by ‘O’ and ‘X’, respectively.

### 2.2. Analytical model validation

The analytical model was evaluated by comparison with *in vitro* experiments conducted under varying injection flow rates and tilt angles. The experimental results are overlaid on the analytical phase diagram in figure 2*b*, enabling direct comparison between predicted and observed filling behaviour. The analytical model captured the transition between successful and failed filling regimes, particularly at low Re numbers (Re < 10). Deviations were observed at higher Re numbers, where experimental cases exhibited successful filling despite analytical predictions of failure.

### 2.3. Fabrication of ideal aneurysm model

Aneurysm models were constructed in SpaceClaim R2024 (SpaceClaim Corporation, Concord, MA, USA), and 3D-printed using a Prusa MK3S+ FDM printer with acrylonitrile butadiene styrene (ABS) filament. The aneurysm morphology for the idealized model was based on previous studies [15,16] with a neck size of 3.5 mm representing a medium-sized aneurysm neck (figure 3*a*). The 3D-printed model was used as a mould into which silicone (Elastosil, Wacker) was poured and allowed to cure overnight. The final fabrication step involved immersing the model in acetone for several days to dissolve the ABS material. To ensure a hydrophilic surface, a 1% (w/v) bovine serum albumin (BSA) solution in PBS was used to coat the inner geometry. This was achieved by washing the inner surface of the model, filling it with the solution and incubating it at 37°C for 1 h, followed by rinsing with PBS.

**Figure 3.**
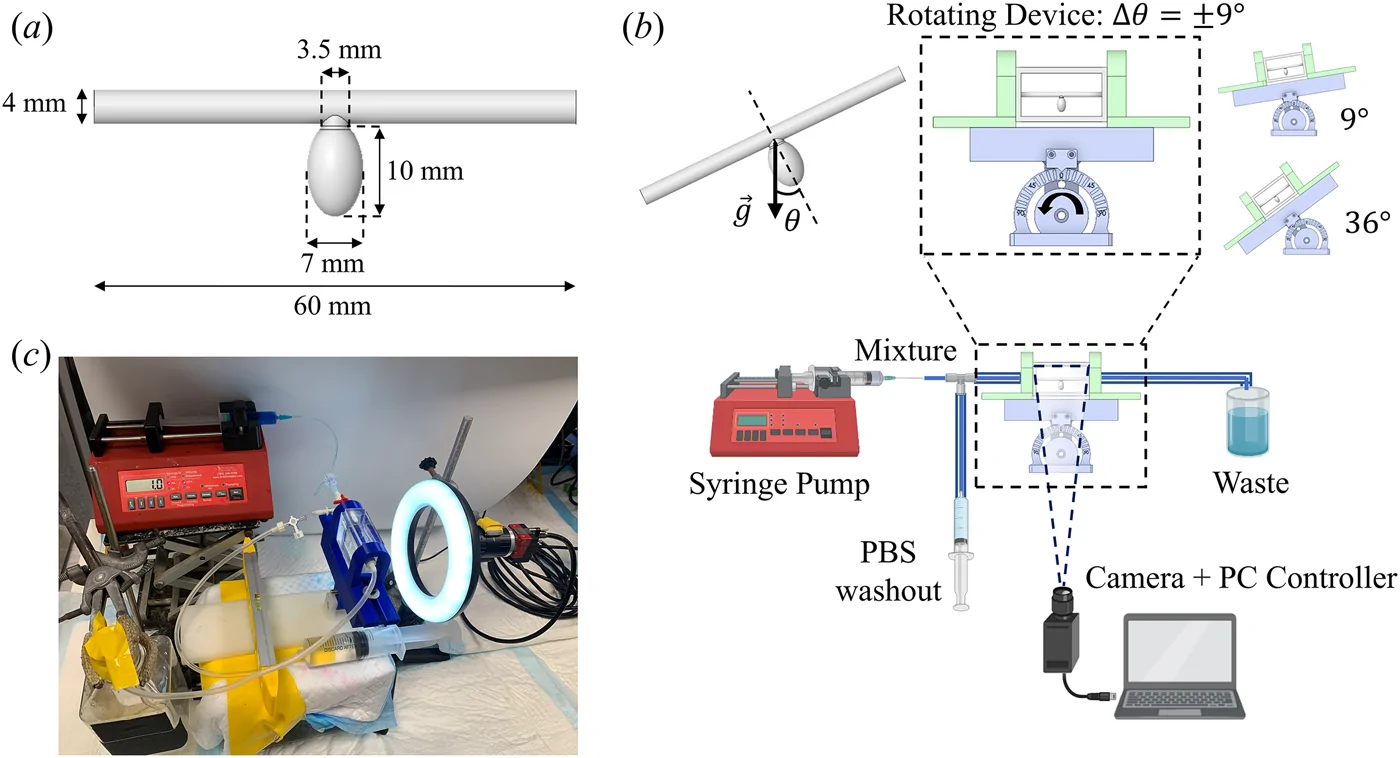
Experimental set-up and ideal aneurysm model parameters. (*a*) Schematic showing the ideal aneurysm model geometrical parameters. (*b*) Schematic description of the experiment set-up and model geometry (constructed via Bio-Render™). (*c*) Image of the experimental set-up.

### 2.4. Experimental flow system, image acquisition and analysis

The flow system (figure 3*b*,*c*) consisted of a syringe pump connected to a 10 ml syringe, which was further connected to a BTPE 50 catheter (polyethylene tubing, 0.58 × 0.97 mm; Instech Laboratories). The catheter was used to inject a 50/50 (v/v) glycerol/PBS mixture, with a density of 1155 kg m^−^³ and a dynamic viscosity of 5.5 mPa s. The catheter was connected to a two-way valve, with a secondary inlet from a 50 ml syringe containing PBS/distilled water used for flushing the model between experiments. The model was securely mounted within a 3D-printed polylactic acid (PLA) rotation device, which ensured stability and allowed control of the rotation angle throughout the experiment. The model was tilted from 0° to 72° in increments of 9°, and for each angle, multiple flow rates were tested. A Sony IMX226 colour camera (1/1.7“, 4072 × 3046 resolution) equipped with a 16 mm lens was used to capture images during the experiments and was controlled via a PC. Time-lapse images were captured during the experiments and analysed using ImageJ. In each image, the region of interest (ROI) corresponding to the aneurysm was manually selected.

Subsequently, images were converted to greyscale, and a threshold was applied to quantify the fraction of black pixels within the ROI, defined as the normalized projected area (NPA) of the liquid filling the aneurysm. NPA values were obtained from images captured throughout the experiment until a steady state was reached. Once a steady state of the injection-driven filling process was reached, the syringe pump was turned off, and additional images were acquired until a second steady state was reached. This secondary steady state resulted from downstream transport of fluid remaining in the upstream artery and from partial outflow of fluid located in the upper region of the aneurysm owing to the model orientation.

### 2.5. Computational fluid dynamics

Numerical analysis was performed on three patient-specific aneurysms that were taken from the Aneurisk database (C0010, C0039 and C0093). Each aneurysm was located in a different region of the Circle of Willis (CoW), as follows: C0010—anterior cerebral artery (ACA), C0039—internal carotid artery (ICA) and C0093—middle cerebral artery (MCA). Each model was refined using commercial software (SpaceClaim R2024), and a BTPE 50 catheter model was added as the injection inlet. All models were meshed using Ansys Meshing® with polyhedral cells, ranging from 850 000 to 1.5 million cells. A refined mesh size was applied to the catheter, artery and aneurysm, and five inflation layers were added along the domain walls. A mesh convergence study was performed (see supplementary material). Prior to the simulations, each model was rotated to align with the gravitational direction that promotes fluid entry into the aneurysm cavity. The domain was prefilled with blood with a dynamic viscosity of 3.5 mPa s and a density of 1060 kg m^−^³. A higher-density fluid (1400 kg m^−^³), with the same dynamic viscosity as blood, was injected from the inlet at a constant rate of 1 ml min^−1^. A laminar flow model was employed, consistent with the low Reynolds number regime (Re ≈ 10–50) and the predominantly stagnant conditions in the domain during injection. Zero-pressure boundary conditions were imposed at all outlets, and no-slip conditions were applied at all walls. A volume of fluid (VOF) method was used to model the interaction between the injected and surrounding fluids, assuming immiscible phases. Simulations were conducted using a pressure-based solver with SIMPLE pressure–velocity coupling. Second-order upwind discretization was applied to the momentum equations, PRESTO! was used for pressure interpolation, and a compressive scheme was employed for the volume fraction. Transient simulations were performed using a first-order implicit time integration scheme. All simulations were carried out using Ansys Fluent 2024 R2.

### 2.6. Geometry and parameter ranges

The study considered both idealized and patient-specific aneurysm geometries. The idealized configuration consisted of a saccular aneurysm connected to a straight parent artery, providing a simplified representation of the aneurysm–artery system. Patient-specific geometries included representative ACA, MCA and ICA aneurysms, all with characteristic dimensions of the order of millimetres (table 1). The investigated conditions followed the parameter ranges defined in the analytical framework. Injection flow rates spanned over two orders of magnitude (O(10⁻¹–10¹) ml min^−1^), while tilt angles varied from near-horizontal to gravity-assisted orientations (O(10¹)°). The film height predicted by the analytical model was of the order of O(10⁻^4^) m, which was comparable to the inner diameter of the catheter used in the experimental injection process. The injected phase consisted of a denser fluid with viscosity of the order of 10^−3^ Pa s, with higher-viscosity cases reaching O(10^−2^) Pa s. The surrounding phase was modelled with properties representative of blood. These configurations enabled systematic evaluation of the coupled effects of geometry, flow conditions and fluid properties on aneurysm filling.

**Table 1.** Geometrical values of the patient-specific models: anterior cerebral artery (ACA), internal carotid artery (ICA) and middle cerebral artery (MCA).

model	mean diameter (mm) [17]	max aneurysm width (mm)*
ACA	2	4.5
ICA	3.6	13.05
MCA	2	10.8

*Note:* *Obtained from computer-aided detection (CAD) measurements.

## 3. Results

### 3.1. Aneurysm filling kinetics

The kinetics of aneurysm filling were evaluated under varying injection conditions using idealized aneurysm models. Figure 4*a* shows the evolution of aneurysm filling (normalized projected area; NPA) over time from a side view for a glycerol/PBS mixture (50/50 v/v) at two tilt angles, *θ*  =  0° and 9°, across the three tested flow rates (0.1, 1 and 10 ml min^−1^). Figure 4*b* shows representative snapshots of the filling dynamics at a fixed tilt angle (9°), highlighting the differences in flow behaviour across injection rates. At the highest tested flow rate (10 ml min^−1^), rapid filling was observed, reaching near-complete filling within approximately 15 s. However, this rapid filling was associated with a drawback: a considerable portion of the fluid bypassed the aneurysm site. This effect was more pronounced at steeper tilt angles. Conversely, at the lowest tested flow rate (0.1 ml min^−1^), stable and gradual filling was achieved without observable fluid bypass. This behaviour persisted across higher tilt angles (up to 72°), indicating the robustness of the controlled filling regime. However, this was accompanied by significantly longer filling times, reaching approximately 2 min at 9° and almost 4 min at 0°, reflecting the reduced gravitational contribution at lower tilt angles. At a fixed injection rate of 1 ml min^−1^, filling time remained approximately constant at 15 s across physiologically relevant tilt angles (9°, 18°, 27° and 36°), indicating a limited influence of orientation on filling kinetics under these conditions (see figure 5; movie S1 in the supplementary material). These results indicate that tilt orientation has a relatively minor effect on filling time compared with injection flow rate. However, the tilt angle is essential for initiating gravitationally driven flow, enabling the initial entry of fluid into the aneurysm cavity. Furthermore, variations in injected mixture properties, including viscosity and density, had minimal impact on filling time. In contrast, a higher-viscosity mixture (approx. 19 mPa s) resulted in approximately 25% increase in filling time across all cases.

**Figure 4.**
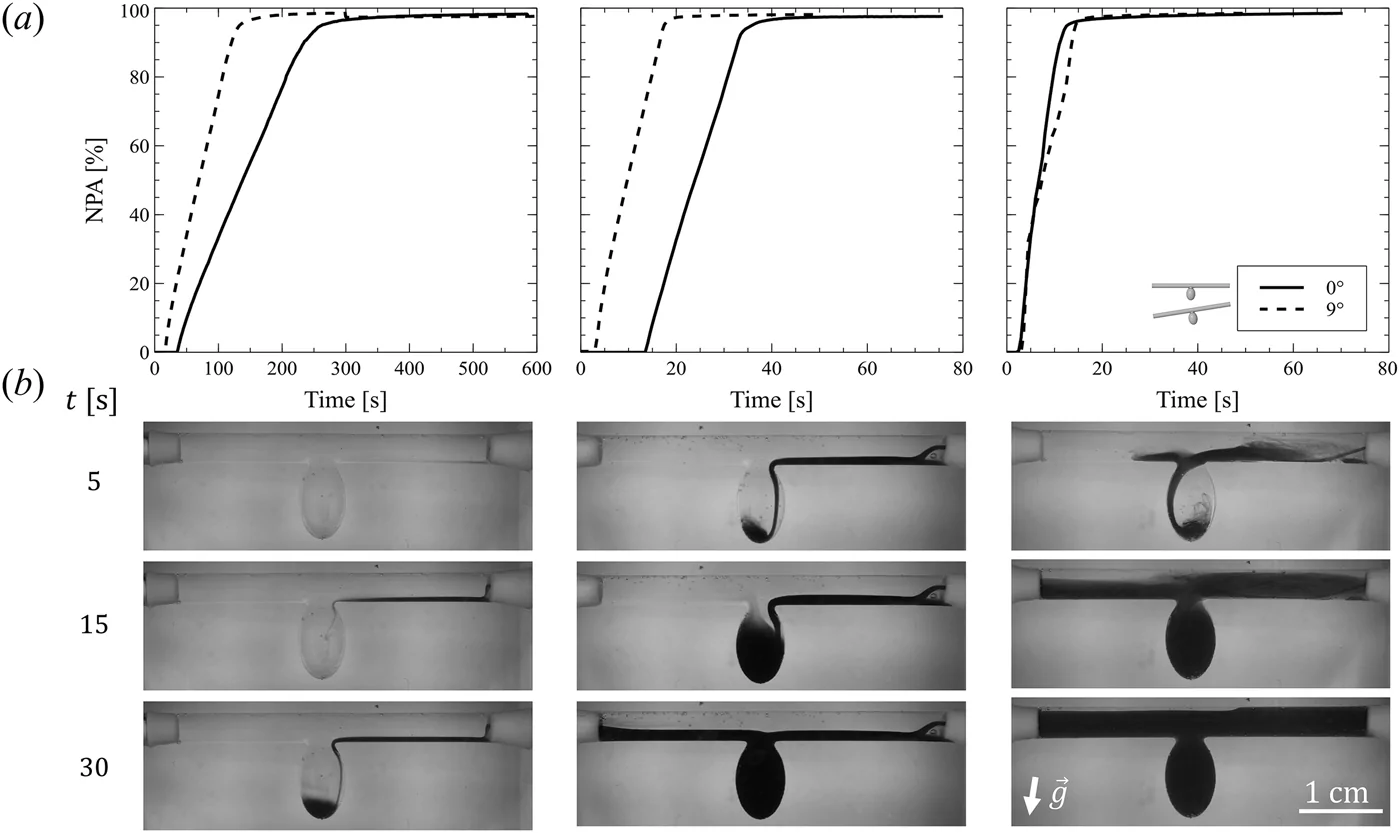
Comparison of aneurysm filling process at three volumetric injection flow rates: 0.1, 1 and 10 ml min^−1^ (columns from left to right, respectively) and for two tilt angle configurations. (*a*) Normalized projected area (NPA) over time for three different flow rates at tilting angles of θ = 0° (solid line) and 9° (dashed line). (*b*) Greyscale images for the 9° tilt angle case. For each flow-rate column, rows represent different time points during the filling process (*t* = 5, 15 and 30 s). For visualization purposes, all experimental images were rotated clockwise to appear horizontal.

**Figure 5.**
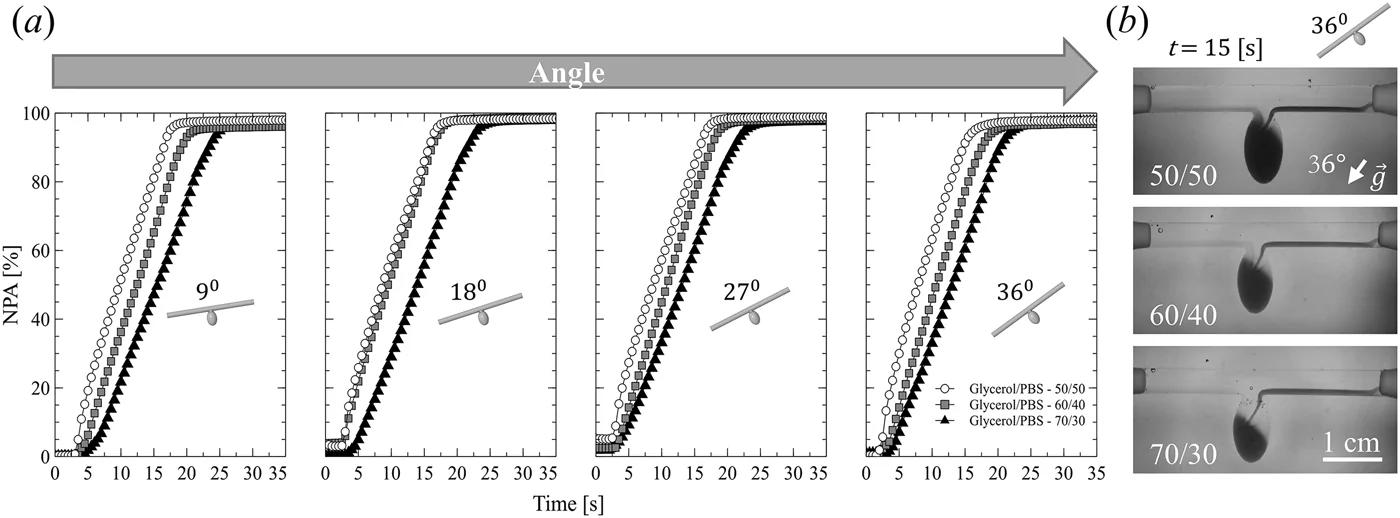
Examination of tilted angle and fluid properties on the filling kinetics. (*a*) Normalized projection area (NPA) over time for four different tilted angles of: 9°, 18°, 27° and 36° for three concentrations of glycerol/PBS mixtures (v/v): 50/50, 60/40 and 70/30. (*b*) Images at *t* = 15 s for all mixtures at a tilted angle of 36°. For visualization purposes, all experimental images were rotated clockwise to appear horizontal.

### 3.2. Patient-specific filling of aneurysms and morphological analysis

Patient-specific CFD simulations were used to evaluate the proposed approach under realistic anatomical conditions. Three representative cases were analysed: the ACA, MCA and ICA, each exhibiting distinct vessel tortuosity and anatomical positioning within the CoW. A distal injection site enabled effective aneurysm filling without requiring direct catheter access to deeper regions of the CoW, while gravity-favourable orientation facilitated fluid entry into the aneurysm. The simulations show substantial aneurysm filling using a denser injected fluid (figure 6), consistent with gravity-driven accumulation within the cavity. However, the time required to achieve sufficient filling varied and was influenced by arterial morphology, injection flow rate and aneurysm configuration. Gravity-favourable orientation was critical for enabling effective aneurysm filling across all models. The ACA model was smaller than the MCA and ICA models. As shown in figure 6, approximately 5 s were required to achieve near-complete filling of the aneurysm cavity in the case of the ACA. Part of the injected fluid entered an adjacent artery, while another portion accumulated in the upper region of the PA. Both effects result from the model's orientation relative to gravity. In contrast, the MCA model required approximately 28 s to fill and exhibited no leakage into an adjacent artery. The ICA model exhibited the longest filling time (approx. 83 s), reflecting its larger aneurysm volume (approx. 2.5 times that of the MCA). Figure 7 shows the progression over time of aneurysm filling alongside the cumulative injected volume normalized by aneurysm volume for each case. The gap between these curves represents the volume of fluid occupying the PA rather than entering the aneurysm. In the ACA case, nearly 200% of the aneurysm volume was injected before full filling was achieved, owing to the small aneurysm size. This effect was less pronounced in the MCA and ICA models, where larger aneurysm volumes reduced relative fluid loss. In the ICA case, a change in slope occurred after approximately 25 s, indicating fluid accumulation within the parent artery.

**Figure 6.**
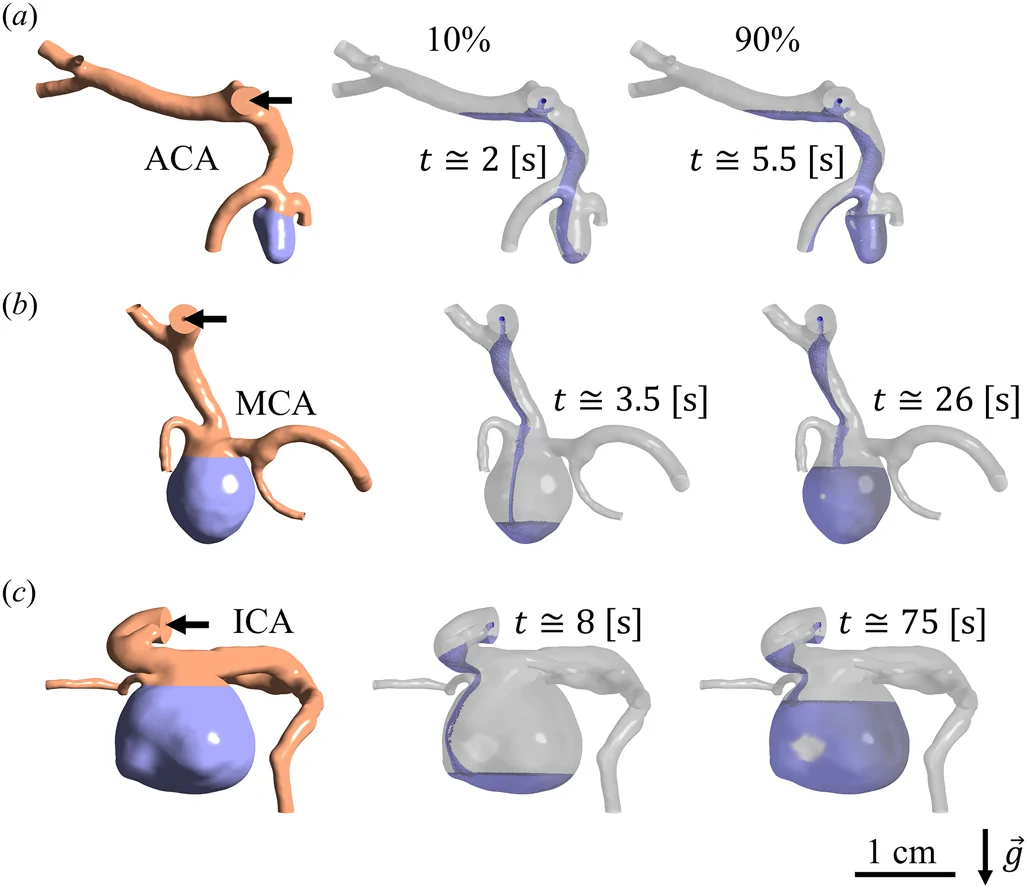
Computational‑fluid‑dynamics (CFD) simulations of a relatively high‑density fluid (1400 kg m^−3^) injected into patient‑specific cerebral‑artery models. The left‑hand column shows three models with an artery and aneurysm coloured with light red and purple, respectively, and a black arrow that indicates the catheter location where the fluid is injected from. (*a*) Anterior cerebral artery (ACA), (*b*) middle cerebral artery (MCA) and (*c*) internal carotid artery (ICA). Gravity‑favoured orientation for an injection rate of 1 ml min^−1^, for each of the three cases (rows) shown at two instantaneous time points linked to distinguish volume filling (columns 10% and 90%). The purple colour in the two right-hand columns represents the iso-volume of the injected liquid with a volume fraction above 0.5.

**Figure 7.**
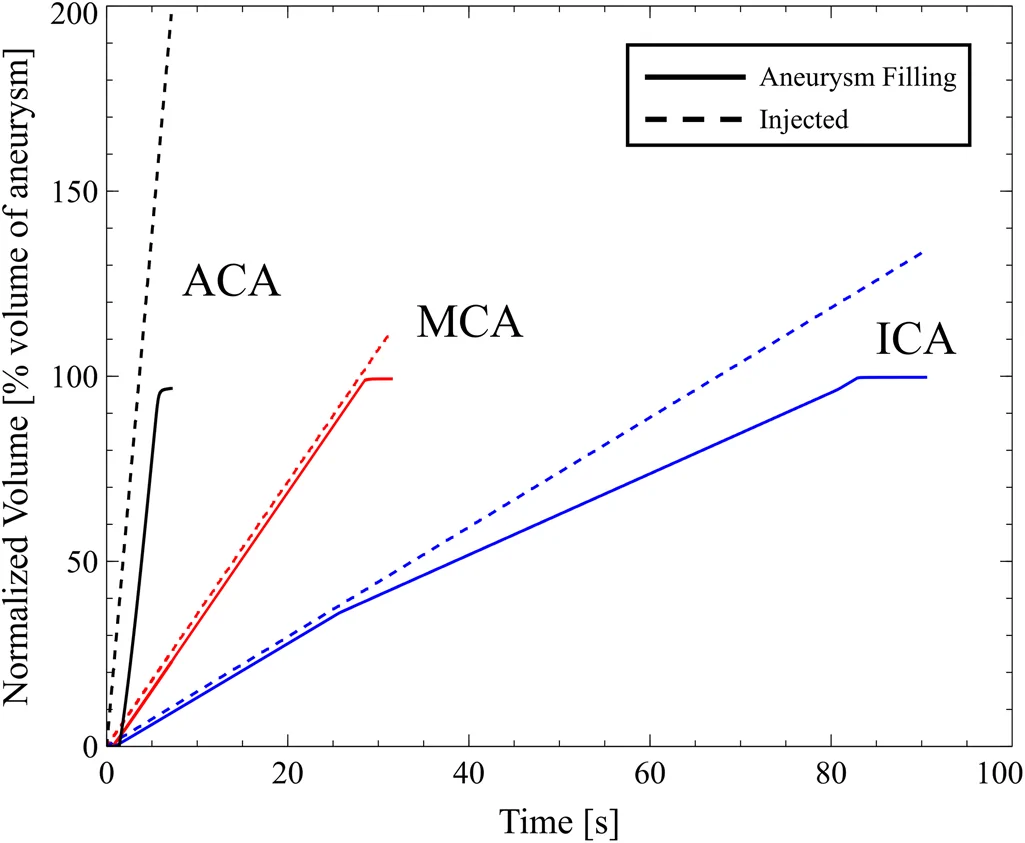
Quantification of normalized volume versus filling time for three different patient-specific aneurysm models. Patient-specific aneurysms: anterior cerebral artery (ACA, black lines), middle cerebral artery (MCA, red lines) and internal carotid artery (ICA, blue lines). Solid lines represent the volumetric filling of the aneurysm, and the dashed line indicates the percentage of aneurysm volume injected during the filling process.

## 4. Discussion

This study demonstrates that gravity-driven flow can enable effective and controlled filling of intracranial saccular aneurysms, governed by the interplay between injection flow rate, fluid properties and geometric orientation. The results show that, under appropriate conditions, gravitational forces can be harnessed to direct fluid into the aneurysm cavity while minimizing bypass into the parent artery. The absence of a consistent anatomical orientation for intracranial aneurysm development [18] indicates that the effectiveness of this mechanism is inherently patient-specific, highlighting the importance of geometry-driven pre-procedure planning. These findings extend previous observations on gravity-driven transport by identifying the conditions under which gravitational forces can be effectively leveraged for targeted intravascular delivery. In this context, gravity-driven flow provides a passive mechanism for guiding fluid motion in complex vascular geometries, with potential implications for minimally invasive cerebrovascular intervention.

### 4.1. Physical mechanism and phase diagram—theoretical versus experimental analysis

Gravity-driven aneurysm filling is primarily governed by the balance between inertial and gravitational forces, with the Fr number emerging as the key parameter controlling the transition between successful filling and bypass. The analytical model describes the interaction between two immiscible fluids on an inclined surface, where a denser injected fluid displaces a quiescent lighter fluid. The resulting flow behaviour is governed by the interplay between inertia, viscosity and gravitational forces, which are characterized by the Re and Fr numbers. The Fr number, which quantifies the relative importance of inertial and gravitational forces, played a role in determining whether injected fluid enters the target region. At high Fr values (corresponding to increased injection rates and steeper inclinations), inertial forces dominated, causing a portion of injected fluid to bypass the target site. In contrast, for Fr < 1, gravitational forces governed the flow, enabling controlled fluid accumulation within the confined region. The phase diagram in figure 2*b* captures the transition between successful and failed aneurysm filling, with analytical predictions consistent with experimental observations at low Reynolds numbers (Re < 10).

However, at higher Re numbers (Re > 20), discrepancies emerged, with experimental cases exhibiting successful filling despite analytical predictions of failure. This behaviour may be attributed to unsteady flow conditions near the injection site, which become more pronounced at higher Re numbers and are not captured by the simplified analytical model. Another possible explanation for this discrepancy may relate to differences in how the velocity magnitude was determined for calculating the Fr and Re numbers between experimental and theoretical analyses. Additional discrepancies may arise from differences in the definition of characteristic velocity between the analytical model and experimental conditions.

In the analytical model, the average velocity across the film cross-section was explicitly calculated using equation (2.3), whereas in the experimental set-up, the velocity was derived based on the catheter's cross-sectional area. Notably, the film height used in equation (2.3) is of the same order of magnitude as the catheter diameter (approx. 10⁻⁴ m).

At low injection rates, the difference in viscosity may contribute to maintaining streamline attachment to the thin film as the heavier fluid also exhibited higher viscosity values. Additionally, differences between experimental and analytical models probably played a role as well. While the analytical model assumes a rectangular two-dimensional cross-section tube, the experimental set-up involved a three-dimensional circular channel and aneurysm model, potentially leading to variations in the thin-film height and affecting flow behaviour at comparable velocities. The proximity between the injection site and the target region posed additional challenges, particularly by preventing the flow from reaching a steady state before interacting with the aneurysm. At higher flow rates (e.g. 10 ml min^−1^), the injected fluid exhibited jet-like behaviour upon impact with the inclined plane, redirecting towards the walls and generating disordered flow patterns (figure 4*b*). Extending the length of the domain upstream of the aneurysm could allow the flow of the injected fluid to stabilize and achieve a fully developed flow regime before reaching the target, thereby improving predictability and reducing bypass events.

The density difference between the two fluids also influenced flow behaviour, affecting settling and accumulation. In these experiments, a normalized density difference (Δ*ρ*  =  (*ρ*_inj_ − *ρ*_surr_)/*ρ*_surr_) of 0.15 was sufficient to induce settling, despite being lower than the typical value for contrast agents (approx. 0.3) [13,19]. This suggests that even moderate density differences can impact gravitational flow dynamics. Although narrow and wide-neck aneurysms are of the same order of magnitude in length, a wider neck is expected to allow greater fluid entry and accumulation, thereby reducing the likelihood of bypassing. This enhanced filling efficiency would also be expected to result in lower Fr values, consistent with the observed flow behaviour. This highlights the role of characteristic length scales in shaping flow behaviour under gravitational influence.

While viscosity differences were expected to contribute to changes in flow dynamics, their effect was found to be minimal within the range examined (figure 5). The impact of viscosity can be further analysed using the volumetric flow rate expression for the denser fluid. Although analytical calculations predict successful aneurysm filling based on the trajectory of the highest streamline entering the cavity, this was not consistently observed in experimental cases. In experimental observations, the jet of the heavier fluid reached the aneurysm site; its impact location often caused only partial entry into the aneurysm, with some of the fluid bypassing the cavity before complete filling occurred. In certain cases, fluid even began to spill over before the aneurysm was fully filled. This discrepancy may be attributed to the momentum of the jet upon impact, which redirected part of the flow away from the aneurysm cavity, thereby limiting effective accumulation. For these cases, the experimental definitions of successful and failed filling were determined by visual inspection, based on whether most of the aneurysm volume was completely filled before spilling occurred. Future work should incorporate the effects of interfacial tension and finite miscibility. Although glycerol and water are considered miscible with one another and therefore operate in a regime characterized by an effective interfacial tension (10^−2^–10^−3^ mN m^−1^) [20], at low injection rates, a well-defined interface was observed between the mixture and the prefilled domain (figures 4*b* and 5*b*). Because experimental measurements rely on contrast-enhanced imaging, the potential influence of molecular diffusion can be assessed through the Schmidt number, defined as the ratio of momentum diffusivity to mass diffusivity. For glycerol–water systems at room temperature, the mass diffusion coefficient is of the order of 10^−9^ m^2^ s^−1^ [21] whereas the kinematic viscosity is of the order of 10^−6^ m^2^ s^−1^, resulting in Schmidt numbers of the order of 10^3^ (Sc ≫ 1). This indicates that momentum transport is much faster than molecular diffusion. As a result, mass transfer during the injection process is dominated by advective transport and interfacial motion rather than diffusion, and molecular mixing is not expected to significantly affect the observed aneurysm filling dynamics within the experimental time scales considered in this study. Time-dependent modelling is also suggested to improve the accuracy of theoretical predictions and enhance the applicability of these findings to clinical practice. Such refinements would aid in optimizing fluid injection strategies for gravity-assisted endovascular interventions, ensuring greater precision and reducing the risk of filling overdose or bypassing the aneurysm. These findings indicate that maintaining a low Fr number is critical for achieving controlled aneurysm filling, while deviations from idealized conditions (e.g. unsteady flow and three-dimensional effects) explain discrepancies between theory and experiments.

### 4.2. Aneurysm filling kinetics—effect of flow rate and tilt angle

Building on the physical insights described in §4.1, injection flow rate emerges as a key parameter governing aneurysm filling dynamics. Lower flow rates promote controlled fluid accumulation within the aneurysm, whereas higher flow rates increase inertial effects and lead to partial bypass of the injected fluid. The analysis considered injection flow rates of 0.1, 1 and 10 ml min^−1^ to assess their impact on the successful filling of the aneurysm. The results reveal a clear trade-off between the speed of filling and the risk of fluid bypassing the aneurysm site. At the highest flow rate (10 ml min^−1^), increased inertial effects led to unstable flow behaviour and promoted bypass of the injected fluid, reducing filling efficiency. This behaviour was strongly influenced by the orientation of the catheter within the domain. Owing to the low-Reynolds-number regime of the injection, the flow was viscosity dominated and did not exhibit classical jet-like expansion; thus, geometric confinement near the wall has a limited effect on the overall filling dynamics. Catheter orientation presents a challenge in achieving consistent flow alignment; this task may be even more complicated to achieve during clinical intervention. The injected fluid entered the domain and did not slowly flow down along the inclined artery model, but rather it impacted the wall and was redirected upwards, thus leading to fluid bypassing the aneurysm site (figure 4). This effect may be mitigated by selecting the injection site to be further away from the aneurysm site, letting the flow reach a steady state while flowing down an inclined artery. However, such conditions may not always be anatomically feasible. Therefore, lower flow rates are favourable. Lower flow rates enabled a successful and stable filling. For the slowest flow rate tested (0.1 ml min^−1^), the filling process was stable and gradual but took 10 times longer than for a flow rate of 1 ml min^−1^, 120 s compared with 15 s for a tilt angle of 9°. Among the flow rates tested, 1 ml min^−1^ emerged as the optimal rate. It provided a balance between avoiding the risk of fluid bypass while ensuring a short enough filling time (less than 1 min). This time is in line with the often-used inflated balloon upstream that can only be used to block flow in brain vessels for several minutes to avoid risking significant patient harm [22].

### 4.3. Patient-specific scenarios—morphological influence on aneurysm filling

Extending these findings to realistic anatomies, patient-specific geometry and catheter positioning emerge as key factors governing aneurysm filling behaviour. Under realistic conditions, the morphological configuration of arteries and aneurysms substantially influenced the dynamics of fluid filling. In the three patient-specific cases examined, pre-orienting the models enabled successful filling of the aneurysm without requiring direct access to the aneurysm site. The orientation of the catheter, which was positioned before each simulation based on an assumed optimal configuration, was critical in determining whether adjacent arteries were unintentionally affected during the filling process. To minimize such effects, a slower injection rate proved more effective. This is evident in the MCA and ICA cases (figure 6*b*,*c*), where an injection rate of 1 ml min^−1^ resulted in more targeted filling compared with the 5 ml min^−1^ cases shown in figure 8. In the ICA case, the tortuous section of the artery between the injection site and the aneurysm posed a potential challenge for navigation. Consequently, a distal injection site was used (figure 8*a*; supplementary material, movie S2). At this location, the fluid initially accumulated within the artery before flowing into the aneurysm, resulting in a delayed filling process. This delay is reflected in figure 7 for the ICA case, where a change in the slope of the filling curve corresponds to temporary accumulation of the injected fluid within the artery leading to the aneurysm. Furthermore, the specific morphology of the aneurysm and parent artery caused an adjacent proximal artery to fill during injection, further complicating the process. Smaller aneurysm and artery sizes led to shorter filling times, as observed in the ACA case (figure 6*a*) compared with the MCA and ICA cases. Additionally, the orientation of the aneurysm relative to gravity played an important role in directing the flow into the aneurysm while minimizing the risk of filling adjacent arteries. This is illustrated in figure 8*b*, where a rotational analysis of the MCA aneurysm shows that a relatively large tilt angle prevented filling of a downstream adjacent artery compared with the gravity-favoured case. However, an artery close to the injection site was still filled early in the injection in all cases, before the fluid reached the aneurysm, owing to catheter orientation. These findings suggest that gravity-based filling strategies should be tailored to each patient, considering the injection flow rate, catheter location, aneurysm orientation and the physical properties of the injected fluid. As a first step, this study provides valuable insights into gravity-driven aneurysm filling. However, several limitations must be acknowledged. The success of the filling process during aneurysm treatment is influenced by multiple factors that require careful pre-procedure consideration, emphasizing the need for thorough morphological analysis before treatment.

**Figure 8.**
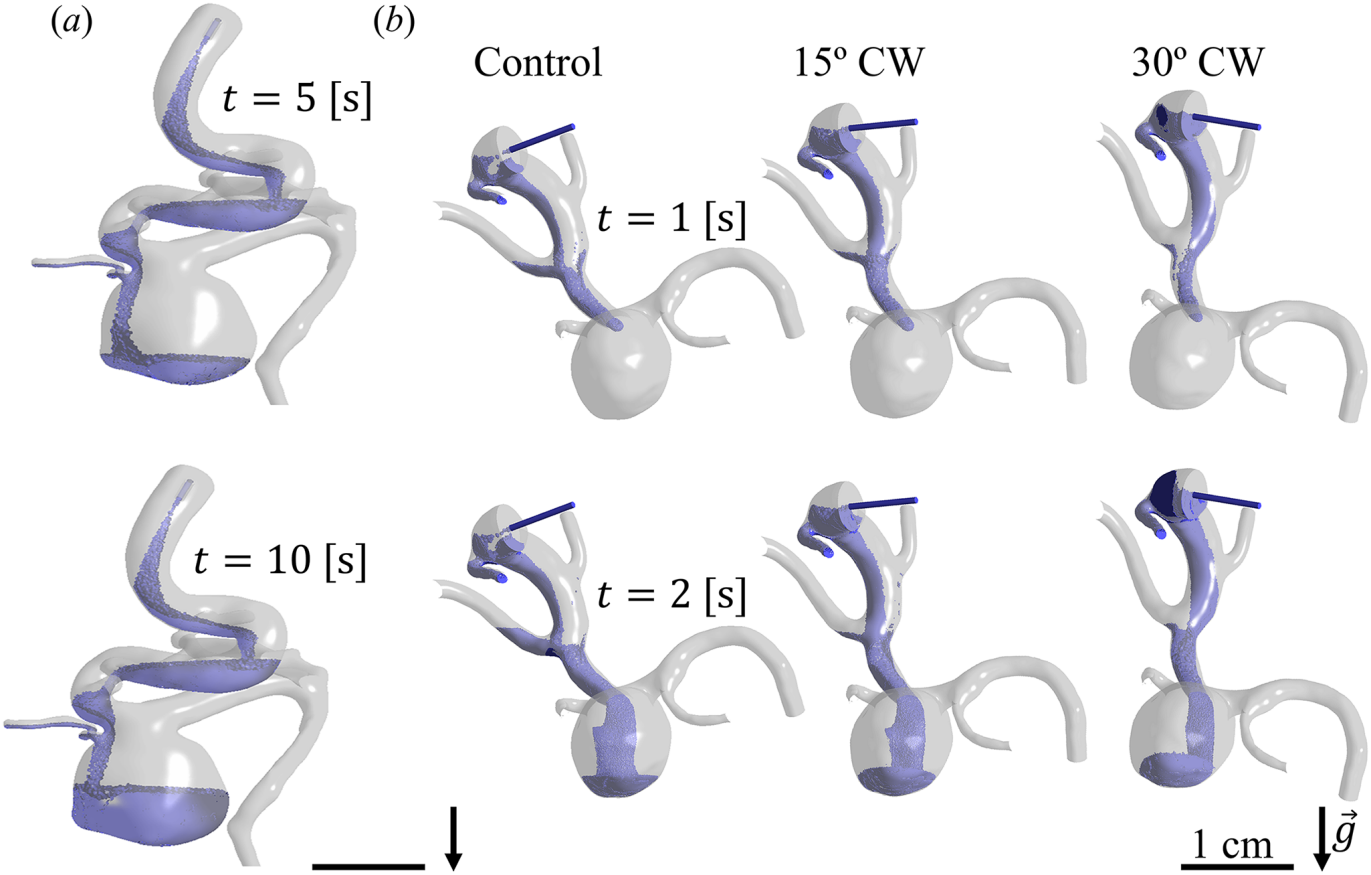
Computational fluid dynamics (CFD) simulation of higher flow rate and rotational analysis of patient-specific aneurysm models. (*a*) Saccular aneurysm attached to the internal carotid artery (ICA) at 5 ml min^−1^ injection flow rate at two different time points: *t* = 5 and 10 s. The model was oriented in the assumed gravity-favoured direction to enhance the filling process used by gravity-driven displacements, and the injection location is relatively distal. (*b*) Rotational numerical analysis of a middle cerebral artery (MCA) aneurysm. Simulation results are shown at two time points, *t* = 1 and 2 s, for a model oriented in a gravity-favoured direction to enhance the filling process, and for two additional configurations rotated clockwise (CW) by 15° and 30°. The fluid was injected into the domain at a flow rate of 5 ml min^−1^ from a relatively distal location.

### 4.4. Translational considerations

From a clinical perspective, gravity-driven liquid filling offers potential both for embolization procedures and for localized therapeutic delivery to the aneurysm site, while requiring careful control over material behaviour during delivery. For embolization, however, the liquid may solidify prematurely during its transit towards the aneurysm or even within the catheter [6]. Such an event could compromise both safety and efficacy, highlighting the need for strategies that allow precise control of solidification. Approaches such as externally triggered solidification or pre-programmed timing mechanisms may help ensure that the liquid reaches the target site before hardening occurs. In addition, when liquids are designed to function as therapeutic carriers, they provide an opportunity to deliver drugs directly to the diseased aneurysm wall, with controlled washout kinetics that may allow transient exposure tailored to treatment objectives. This perspective aligns with the growing interest in biophysical targeting strategies for aneurysms [15] and with recent demonstrations of fluid-based methods that confine therapeutics within the aneurysm cavity [23]. While such clinical translations are beyond the scope of the present study, they point to promising directions for future research and application.

## 5. Conclusions and future work

This study demonstrates the potential of using gravity-driven fluid injections to effectively fill intracranial saccular aneurysms. While gravity-driven injections show promise, their success is highly dependent on careful consideration of aneurysm orientation, vessel morphology and appropriate control of injection parameters. Mapping the pathway from the injection site to the aneurysm enables identification of potential complications and optimization of catheter placement to enhance delivery. Advanced imaging techniques, such as rotational angiography or three-dimensional reconstructions, could provide critical insights into the vascular pathway, aiding in pre-treatment planning [24,25]. Patient-specific CFD modelling can provide a virtual framework to evaluate potential complications and adjust procedural parameters, ensuring more controlled and efficient fluid delivery during treatment. Moreover, angiographic imaging using a contrast agent with similar properties as the injectable biomaterials can be used to examine filling kinetics during the procedure and before treatment with the biomaterial, thereby ensuring safe and effective application of this method. Further patient-specific studies are required to better understand the limitations of the filling process when injecting from a distant location, and to determine optimal patient positioning to facilitate gravity-driven filling. Additionally, this study primarily examined fluid mechanics rather than rheological properties of the injected fluid. Investigating biomaterials with different fluid characteristics, such as injectable shear-thinning solutions, may enhance the effectiveness of this filling technique. Moreover, studies using suitable biomaterials and animal models are also required to further explore specific treatment opportunities.

## Supplementary Material



## Data Availability

The time-lapse accelerated movies generated in this study are available from the Zenodo repository [26]. The three vascular models used in this study were obtained from the publicly available AneuriskWeb repository [27]. Supplementary material is available online [28].
