# Supplementary material for: Gravity-driven flow for intracranial saccular aneurysm filling
Source: J R Soc Interface. 2026 Oct 28;23(243):20250968. doi: 10.1098/rsif.2025.0968 (PMC13714385; doi:10.1098/rsif.2025.0968)
Supplement: Supplementary file 1 [file rsif.2025.0968.zip › rsif.2025.0968/rsif20250968_si_001.docx]

**Gravity-Driven Flow for Intracranial Saccular Aneurysm Filling**

**- Supplementary Materials**

Tirosh Mekler^1^, Maria Khoury^1^, Yevgeniy Kreinin^1^, Ramy Fishler^1^, Nir Natan^1^, Ron Bessler^1^, Alexander Leshansky^2^, Josue Sznitman^1^, Netanel Korin^1,3^

^1^Department of Biomedical Engineering, Technion – Israel Institute of Technology, Haifa, Israel.

^2^Department of Chemical Engineering, Technion – Israel Institute of Technology, Haifa, Israel.

^3^Fischell Department of Bioengineering, University of Maryland, College Park, Maryland, USA.

Figure S1: Fabrication of the idealized aneurysm model. A 3D printed model and the final model after Elastosil molding and dissolving the inner material with acetone.

Table S1: Summary of mixture properties.

| Mixture (Glycerol/PBS) | Density $\boldsymbol{[}\frac{\mathbf{kg}}{\mathbf{m}^{\mathbf{3}}}\boldsymbol{]}$ | Dynamic Viscosity $\boldsymbol{[}\boldsymbol{mPa\cdot s}\boldsymbol{]}$ |
| --- | --- | --- |
| 50/50 | 1154$\pm$10 | 5.5$\pm$0.1 |
| 60/40 | 1174$\pm$10 | 9.8$\pm$0.2 |
| 70/30 | 1188$\pm$10 | 19.8$\pm$0.4 |

# Spilling Effect

A noteworthy effect that was observed was the spilling of fluids post-filling (see Figure S2A). This behavior is expected given the interplay between gravity, fluid inertia, and the aneurysm’s geometric constraints. After achieving a steady-state filling of the aneurysm, the injection was stopped, and a new steady-state condition was reached as a portion of the mixture drained out from the aneurysm due to spilling. In Figure S2B, the spilling effect is shown for four different tilt angles. Models subjected to high tilt angles (>27°) exhibited reduced volume filling after reaching the new steady state. However, significant filling (>92%) was still achieved for aneurysms with tilt angles less than 27°, indicating that while the spilling effect reduces the contained volume, the procedure remains effective within certain physiological limits.

**Figure S2:** **Spilling effect for different tilt angles.** (A) Images after reaching steady state for injection and spilling at tilt angles of 18° and 36°. (B) The loss in the normalized projected area (NPA) values between the steady state during filling and the NPA post spilling over several tilt angles (N=3, * p<0.05, ** p<0.01 and *** p<0.001). (All experiments images were visually tilted back to appear horizontally).

Figure S3: Application with embolic agent analog. (a) Snapshot of an embolic agent analog (UV crosslinking resin) at a tilted angle of 27°. (b) Streaklines of injected particles inside the model: (i) without embolization, particles appear to flow in the parent artery and enter the aneurysm and (ii) streaklines of injected particles with an embolized aneurysm. Particles are flowing in the parent artery and not entering the aneurysm while an inclined sealing is appearing due to the crosslinking taking place while model is still tilted. (All experimental images were rotated clockwise to appear horizontally).

# Mesh Convergence Test

In order to increase the credibility of the three-dimensional CFD simulations, a mesh convergence test was performed on the smallest patient-specific model, under the assumption that, when using the same meshing protocol for all models examined in this study, the smallest model would be the most strongly affected by mesh resolution. For this test, three different mesh grids were generated with the following polyhedral cell counts: coarse, 4.48×10⁵; medium, 8.51×10⁵; and fine, 1.73×10⁶ for ACA model (C0010). The medium mesh was used for all simulations presented in this study. As shown in Figure S4A, the volume fraction of the drug inside the aneurysm over time does not exhibit significant differences among the three mesh grids. In addition, Figure S4B shows that the velocity magnitude profiles follow the same trend, with a relative error in the local maximum velocity magnitude between the medium and fine mesh of approximately 10%, as summarized in Tables S2 and S3. Additionally, Tables S4 and S5 present the number of polyhedral cells in each mesh used for the mesh convergence test and in each model, categorized by artery and aneurysm regions respectively.

Figure S4: A mesh convergence test was performed on the smallest patient-specific case (C0010) using three different mesh resolutions (coarse, medium, and fine). (a) Drug volume fraction inside the aneurysm space over time for the three grids tested. (b) Velocity magnitude profiles along the red line at each of the three cross-sections shown in the model at two different time points (t_1_ = 2s and t_2_ = 6s).

Table S2: Maximum values of velocity magnitudes at each line used in the mesh convergence test and their relative error to the Fine grid at t_1_ = 2s.

| Location | Coarse [m/s] | Coarse RE [%] | Medium [m/s] | Coarse RE [%] | Fine  [m/s] |
| --- | --- | --- | --- | --- | --- |
| I | 0.03 | 1.8 | 0.03 | 7.9 | 0.03 |
| II | 0.02 | 4.4 | 0.02 | 10.7 | 0.02 |
| III | 0.03 | 2.6 | 0.03 | 2.4 | 0.04 |

Table S3: Maximum values of velocity magnitudes at each line used in the mesh convergence test and their relative error to the Fine grid at t_2_ = 6s.

| Location | Coarse [m/s] | Coarse RE [%] | Medium [m/s] | Coarse RE [%] | Fine  [m/s] |
| --- | --- | --- | --- | --- | --- |
| I | 0.03 | 4.2 | 0.03 | 11.6 | 0.03 |
| II | 0.03 | 5.1 | 0.02 | 13.2 | 0.03 |
| III | 0.03 | 2.2 | 0.03 | 5.8 | 0.04 |

Table S4: Number of polyhedral cells in each C0010 model’s mesh grid divided by zones: artery and aneurysm.

| Zones | Coarse | Medium | Fine |
| --- | --- | --- | --- |
| Artery | 292,625 | 370,116 | 595,639 |
| Aneurysm | 124,611 | 166,207 | 829,280 |

Table S5: Number of polyhedral cells in each model divided by zones: artery and aneurysm.

| Zones | C0010 - ACA | C0039 - ICA | C0093 - MCA |
| --- | --- | --- | --- |
| Artery | 370,116 | 358,307 | 377,869 |
| Aneurysm | 166,207 | 346,824 | 800,022 |

**Movie S1:** Time-lapse accelerated movie showing the filling kinetics of a 50/50 (v/v) glycerol/PBS mixture at four different tilt angles (9°, 18°, 27°, and 36°) with an injection flow rate of 1 mL/min.

## Movie S2: Computational fluid dynamics (CFD) simulation of the filling process in a patient-specific internal carotid artery (ICA) aneurysm at an injection flow rate of 5 mL/min. Theoretical Model

Consider two immiscible fluids with the following properties:

- **Fluid 1**: density $\rho_{i}$ and dynamic viscosity $\mu_{i}$, the injected fluid.
- **Fluid 2**: density $\rho_{c}$ and dynamic viscosity $\mu_{c}$, the continuous phase.

Fluid 1 is injected through a distant catheter inlet into a domain initially filled with quiescent fluid 2. The domain is modeled as an inclined pipe with a rectangular cross-section tilted by an angle $\theta$ relative to the horizontal axis (see Figure 3A). The injected heavier fluid 1 flows down the plate due to gravity, driving the flow of the lighter fluid 2 due to viscosity. Once a steady-state flow is established, the objective is to determine the velocity profile and flow rate of
fluid 1.

To achieve this, we solve the two-dimensional Navier-Stokes equations for both fluids in the X-Y coordinate system, as illustrated in Figure 2A.

Assuming steady state condition and incompressible flow with two immiscible liquids we approximate the stream of the heavier fluid induced by the gravity-driven laminar flow of a liquid film of height $h$ along an inclined plate with the tilt angle $\theta$ with respect to the horizon. We can write the momentum equation in the x direction in general for both fluid ($j=1,2$):

$$0=-\frac{\partial p}{\partial x}+\mu_{j}\frac{\partial^{2}u_{j}}{\partial y^{2}}+K_{j}g\sin\theta$$

(S1)

where ${K_{1}=\rho}_{i}-\rho_{c}>0$ and $K_{2}=0$. From the boundary conditions of no-slip at the upper and bottom surface, velocity and tangential stress continuity at the liquid-liquid interface at $y=h$, the constant (back) pressure gradient $\left( {\partial p}/{\partial x}>0 \right)$is found from the requirement of zero net flow rate of the displaced (lighter) fluid 2, owing to the upstream blockage of the flow.

The average velocity of the heavier fluid 1 in the film of thickness $h$ and the lighter fluid 2 co-flowing down the slit of height $H$ are found from the solution and they read:

$$\bar{V}_{1}=\frac{gh^{2}\Delta\rho\sin\theta}{{3\mu}_{i}} \frac{\left( \beta-1 \right)\left( \beta+\kappa-1 \right)}{\left( \left( \beta-1 \right)^{2}+\kappa\left( 4\beta-1 \right) \right)}=\frac{gh^{2}\Delta\rho\sin\theta}{{3\mu}_{i}}f\left( \beta,\kappa\right) , \bar{V}_{2}=0$$

(S2)

where $\beta=H/{h>1}$ and$\kappa={\mu_{c}}/{\mu_{i}}$ is the viscosity ratio. This formulation is developed for a 2D flow, disregarding the side walls and refers to a shallow and wide film ($h\ll H$). The solution in Eq. S2 can be rewritten as:

$$\frac{\mathrm{Re}}{\mathrm{Fr}^{2}}f\left( \beta,\kappa\right)=\frac{3}{\epsilon}\cot\theta$$

(S3)

where$\mathrm{Re}={\bar{V}_{i}\rho_{i}h}/{\mu_{i}}$ is the corresponding Reynolds number (of fluid 1), $\epsilon=h/{L_{n}}$ and $Fr={\bar{V}_{i}}/{\sqrt{\delta g\cos\theta L_{n}}}$ stands for the modified Froude number with $\delta={\Delta\rho}/{\rho_{c}}$.

Let us assume that the origin of the coordinate system is located at the interface separating the two fluids and x axis is oriented along the interface, as shown in Figure 2A. We next assume that beyond the edge of the aneurysm cavity the stream of the heavy fluid is falling as a free jet under gravity (i.e., neglecting the stress associated with the outflow of the lighter fluid). The corresponding equation of the (ballistic) motion of the jet is described by the 2^nd^ order ODE’s:
$\ddot{y}=-\delta g\cos\theta, \ddot{x}=\delta g\sin\theta$, with the initial conditions on the (mean) velocity of the jet at the edge of the cavity (at the origin): $\dot{x}\left( 0 \right)=\bar{V}, \dot{y}\left( 0 \right)=0$. The resulting jet trajectory is described in the parametric form by:

$$x\left( t \right)=\frac{\delta g\sin\theta}{2}t^{2}+\bar{V}t, y\left( t \right)=-\frac{\delta g\cos\theta}{2}t^{2}$$

(S4)

where$t$ is the time. The parabolic shape of the gravity jet in Eq. (S4) can be rewritten in the explicit form, expressing $t$ from the 2^nd^ equation for $y(t)$ and substituting it into the 1^st^ equation for $x(t)$:

$$x\left( y \right)=-y\tan\theta+\bar{V}\sqrt{\frac{-2y}{\delta g\cos\theta}}$$

(S5)

Denoting the following relations: $\xi=x/{L_{n}}$ and $\eta=y/h$, which are the distances scaled with the aneurysm neck and the film thickness respectively we obtain:

$$\xi=-\epsilon\eta\tan\theta+\mathrm{Fr}\sqrt{-2\eta\epsilon}$$

(S6)

It can be readily seen that the shape of the jet for given $\epsilon$ is a sole function of $\mathrm{Fr}$and the tilt angle $\theta$. We can now define (similar to what is shown in the manuscript) a successful and failure filling whether all the streamlines fall into the aneurysm, or a single streamline passed the aneurysm neck length respectively. Obviously, the critical jet trajectory $\eta\left( \xi_{cr} \right)$ separating successful and failure filling is a sole function of $\mathrm{Fr}$, which itself depends on the flow rate, the density contrast, the tilt angle and the size of the aneurysm neck). The mathematical condition for this critical separatrix dividing success and failure will then be $y_{cr}=-h$ and $x_{cr}=L_{n}$ which corresponds to the dimensionless values $\xi_{cr}=1$ and $\eta_{cr}=-1$. Therefore, we obtain the following equation for the critical Fr as a function of $\theta$ and $\epsilon$:

$$\mathrm{Fr}_{\mathrm{cr}}=\frac{1-\epsilon\tan\theta}{\sqrt{2\epsilon}}$$

(S7)

Substituting the Eq. S7 into Eq. S3 yields the relation between the critical Re and Fr numbers:

$$Re_{\mathrm{cr}}=\frac{3Fr_{\mathrm{cr}}^{2}\cot\theta}{\epsilon f\left( \beta,\kappa\right)}$$

(S8)
